# Automatic recording of rare behaviors of wild animals using video bio-loggers with on-board light-weight outlier detector

**DOI:** 10.1093/pnasnexus/pgad447

**Published:** 2024-01-16

**Authors:** Kei Tanigaki, Ryoma Otsuka, Aiyi Li, Yota Hatano, Yuanzhou Wei, Shiho Koyama, Ken Yoda, Takuya Maekawa

**Affiliations:** Graduate School of Information Science and Technology, Osaka University, Suita, 565-0871 Osaka, Japan; Graduate School of Information Science and Technology, Osaka University, Suita, 565-0871 Osaka, Japan; Graduate School of Information Science and Technology, Osaka University, Suita, 565-0871 Osaka, Japan; Graduate School of Engineering Science, Osaka University, Toyonaka, 560-8531 Osaka, Japan; Graduate School of Frontier Biosciences, Osaka University, Suita, 565-0871 Osaka, Japan; Graduate School of Environmental Studies, Nagoya University, Nagoya, 464-8601 Aichi, Japan; Graduate School of Environmental Studies, Nagoya University, Nagoya, 464-8601 Aichi, Japan; Graduate School of Information Science and Technology, Osaka University, Suita, 565-0871 Osaka, Japan

**Keywords:** animal behavior, bio-logging, rare behavior, outlier detection, AI on animals

## Abstract

Rare behaviors displayed by wild animals can generate new hypotheses; however, observing such behaviors may be challenging. While recent technological advancements, such as bio-loggers, may assist in documenting rare behaviors, the limited running time of battery-powered bio-loggers is insufficient to record rare behaviors when employing high-cost sensors (e.g. video cameras). In this study, we propose an artificial intelligence (AI)-enabled bio-logger that automatically detects outlier readings from always-on low-cost sensors, e.g. accelerometers, indicative of rare behaviors in target animals, without supervision by researchers, subsequently activating high-cost sensors to record only these behaviors. We implemented an on-board outlier detector via knowledge distillation by building a lightweight outlier classifier supervised by a high-cost outlier behavior detector trained in an unsupervised manner. The efficacy of AI bio-loggers has been demonstrated on seabirds, where videos and sensor data captured by the bio-loggers have enabled the identification of some rare behaviors, facilitating analyses of their frequency, and potential factors underlying these behaviors. This approach offers a means of documenting previously overlooked rare behaviors, augmenting our understanding of animal behavior.

**Significance Statement**

The development of an artificial intelligence (AI)-enabled video bio-logger is presented to overcome the challenge of observing rare behaviors exhibited by wild animals. Once the biologists attach the video bio-logger to a target animal, the bio-logger automatically records rare animal behaviors in the wild that have not been observed before. The videos recorded by the bio-logger can be used by biologists to propose a new hypothesis by analyzing the videos in conjunction with associated sensor data. We believe that this study is the first attempt to create an autonomous AI-based naturalist that works on a bio-logger to seek out interesting animal behaviors on behalf of biologists in the wild environment.

## Introduction

In animal behavior, anecdotes or observations of infrequent behavior are often considered important (1) because they can generate novel hypotheses and stimulate new avenues for behavioral, ecological, and evolutionary studies (2). However, owing to their rarity, these behaviors may not even be noticed in the natural field or may not be documented with sufficient sample sizes to enable statistical analyses (3). Also, investigating rare behaviors may often result in overinterpretation, depending on the sample size (4). Although long-term observation is the only way to obtain reliable samples of rare behaviors, it is time-consuming and often impossible in harsh environments such as the sea. Animal-borne data loggers have been utilized to record long-term behavioral data from many individual animals including acceleration and Global Positioning System (GPS) data, which are used to quantify the animal behaviors from various aspects, e.g. calculating a statistic related to energy expenditure from acceleration data (5); detecting area restricted search behaviors from GPS data (6).

The collected long-term data potentially contain sensor readings corresponding to rare behaviors related to their body movements and locomotion. However, determining which sensor readings are associated with rare behaviors and identifying the nature of these behaviors only from the readings is challenging. Although animal-borne video loggers (i.e. video bio-logging) offer insight enabling to identify and understand rare behaviors (7), video bio-logging captures only a few hours of an individual's behavior owing to limited battery capacity (8), resulting in insufficient video recordings for finding or investigating rare behaviors. Thus, new methodologies for video-based bio-loggers are required to target rare events, although paradoxical, over an extended period in order to overcome behavioral rarity.

Simple rule-based bio-logging approaches relying on manually determined thresholds (such as the minimum depth and minimum magnitude of acceleration) (9–14) have been used to address the aforementioned issues associated with video bio-loggers. The notable examples are: recording foraging dives of seabirds when a depth sensor measures a depth beyond 2 m (9); recording feeding events of seals by detecting head or jaw motions with an acceleration amplitude threshold of 0.3 g (11). In addition, a supervised machine-learning-based bio-logger developed by our research group automatically recognizes animal behaviors in real-time using low-cost sensors, such as an acceleration sensor, under the concept of artificial intelligence (AI) on Animals (AIoA) (15). Only when a machine-learning model implemented on the bio-logger detects a target behavior, the bio-logger activates its camera to capture the target behavior, enabling conditional activation of the high-cost camera. A machine-learning model that relies on supervised learning is trained in advance on sensor data with manual annotations of target behaviors. For example, the foraging behaviors of seabirds were detected by their characteristic patterns in acceleration data. However, because these rule-based and supervised machine-learning-based methods have been developed to capture target behaviors envisaged by researchers in advance, they cannot be applied to capture unexpected infrequent behaviors that we have not observed before.

**Fig. 1.**
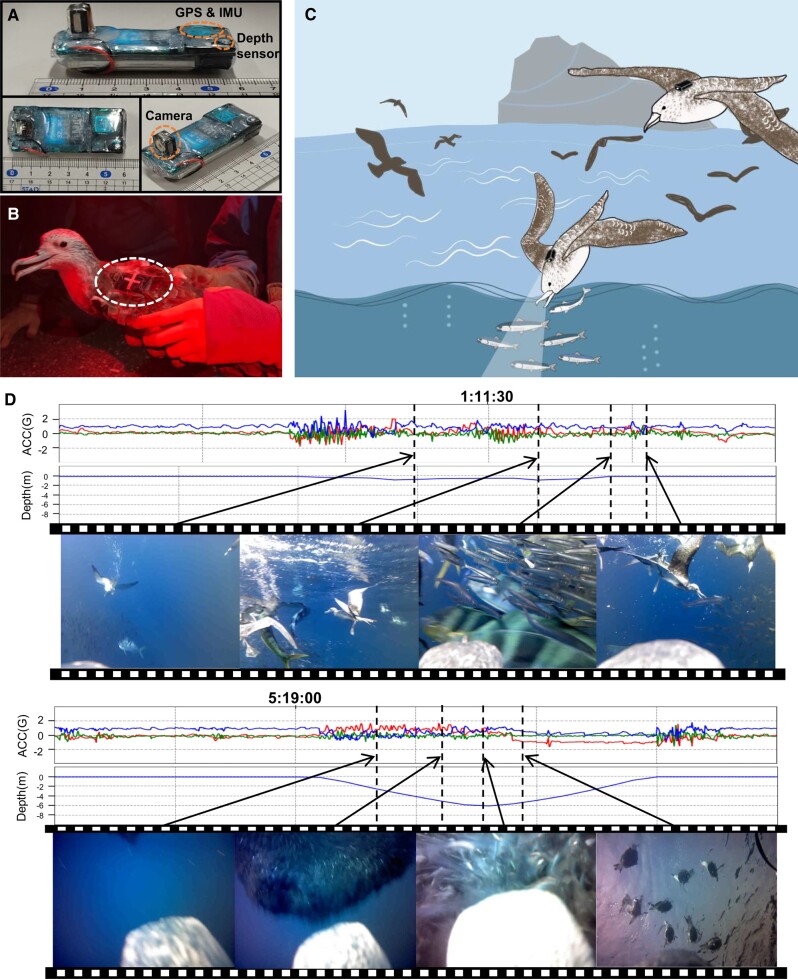
Example use of our bio-loggers for rare event detection and recording. A) A bio-logger was used in this study. B) Bio-logger attached to the back of a streaked shearwater. C) The bird with the bio-logger is released and freely moves in a wild environment. The bio-logger automatically records videos of interesting events without supervision by researchers. D) Example frames and sensor data collected by our bio-logger showing foraging behaviors of streaked shearwaters. See also Movie S1.

To capture rare behaviors without the supervision of researchers, we leveraged unsupervised learning,^a^ which enables us to automatically find patterns from raw sensor data without manual annotations. Although unsupervised clustering techniques have been adapted to offline analysis of animal behavioral data (16, 17), these techniques, which are tailored to identifying major behavior clusters, cannot be employed to identify rare behaviors. Therefore, we define rare behavior as an outlying event and leverage unsupervised outlier detection techniques to automatically find rare behaviors of an animal from the readings of low-cost sensors to record videos of the behaviors. Because rare animal behaviors detected by our approach are considered unusual outlying events, the probability with which the recorded videos capture behaviors that we have never witnessed before is high. Therefore, after attaching the proposed bio-logger to an animal (Fig. 1A and B) and subsequently releasing the animal, the bio-logger automatically records videos containing interesting animal events in a wild environment without supervision by researchers (Fig. 1C). The bio-logger then delivers the videos with sensor data to researchers (Fig. 1D), enabling the researchers to propose a new hypothesis by analyzing the videos in conjunction with the sensor data.

However, there is a methodological issue that needs to be resolved: because general outlier detection methods (18–20) have high memory and computational costs, it is difficult to run these methods on a low-energy micro control unit (MCU) with limited memory and computational power implemented on the bio-logger. To address this issue, we trained an outlier detector on a computer server using unlabeled data from target animals (which is easier to prepare than labeled data) in advance and then employed knowledge distillation^b^ (21) to construct a lightweight outlier detector as a student model using the original outlier detector as a teacher model. We then implemented the student outlier detector on the bio-logger to detect rare behaviors in real-time.

We applied the proposed method to two case studies to illustrate the applicability and effectiveness of a variety of low-cost sensors: (i) using an accelerometer, one of the most commonly used bio-logging sensors, to capture body movements associated with the rare behavior of streaked shearwaters (*Calonectris leucomelas*) at sea and (ii) using a water depth sensor to record movies of their behaviors in the air–water interface region, which is commonly very difficult to distinguish.

## Materials and methods

### Video bio-logging device

Figure 1A shows the bio-logger used in this study. It is 60 × 26 × 22 mm (length × width × height), has a 3.7 V 600 mAh battery, and weighs ∼23 g (<5% of the body mass of the seabirds in our experiment). The bio-logger was equipped with a video camera, three-axis acceleration sensor, GPS unit, water pressure sensor, thermometer, magnetometer, and illuminometer. A low-energy MCU (ATmega328, 32 KB program memory, 2 KB RAM) on the bio-logger controls the sensors and stores the sensor data in dedicated long-term data storage. The acceleration and water pressure sensors, which were used in the following experiment as low-cost sensors, measured the sensor data at 31 and 1 Hz, respectively. Video data captured by the camera (640 × 480, 15 frame per second [FPS]) were also streamed to dedicated storage after the MCU signaled the start of the recording. The running time of the bio-logger was limited to only 2 h when the camera was always turned on because the mass of the bio-logger (battery) was restricted. Note that video cameras, including those used in our bio-logger, have a delay of several seconds from receiving the start signal to when they can begin recording. Our bio-logger has a 5–7 s delay in recording from sleep mode to save energy, preventing us from recording a moment of outlying behavior that triggers video recording. However, because animals are considered to continue a specific behavioral mode, such as foraging, we can still record the remaining behaviors.

Note that although the MCU has 32 KB program memory, basic functions regarding sensor management and data storage already occupy as much as 95% of the memory, requiring the design of a lightweight outlier detection model. The detailed specifications of the bio-loggers are listed in Table S1.

### On board detection of rare behavior of seabirds: overview

**Fig. 2.**
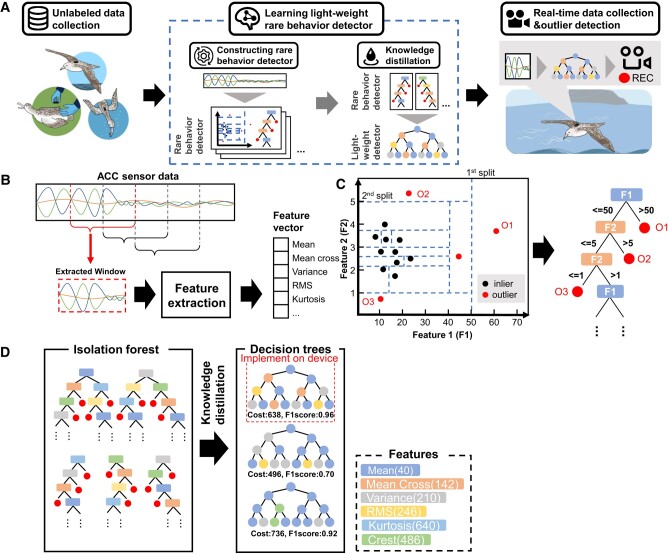
Overview and procedures of our method. A) Method overview. After unlabeled data collection, a rare-behavior detector is constructed on a server computer. Then, a lightweight rare-behavior detector that can run on a bio-logger is constructed from the original rare-behavior detector via knowledge distillation. Finally, the lightweight rare-behavior detector is implemented on a bio-logger and detects outlying behaviors. B) Feature extraction from sensor data and construction of a feature vector using a sliding time window. C) Outlier detection with an isolation tree. The feature space is recursively split by randomly selecting a dimension and a threshold value. The information regarding each split is represented as a tree node in the isolation tree. An outlying feature vector is isolated in fewer splits because it is located far from inliers in the feature space. D) Decision trees are constructed from the isolation forest by knowledge distillation. From the trees, we select a decision tree that fits the available program memory and yields the best recognition performance.

Figure 2A shows an overview of the proposed approach. Our approach is composed of three steps: (i) unlabeled data collection for learning a rare-behavior detector; (ii) learning a lightweight rare-behavior detector; and (iii) real-time data collection and rare-behavior detection. The second step also comprises two substeps: (ii-a) constructing a rare-behavior detector and (ii-b) knowledge distillation. In the first step, unlabeled sensor data from low-cost sensors, such as accelerometer data, are collected from individuals of a target species. When such data are available, we skip this step. In the second step, we define a rare behavior as an outlying event in the sensor data and train an outlier detector as a rare-behavior detector on the unlabeled data on a server computer. We then employ knowledge distillation to construct a lightweight rare-behavior detector as a student model, using the original rare-behavior detector as a teacher model. In the third step, a lightweight rare-behavior detector is implemented on a bio-logger attached to the target animal.

### Constructing rare behavior detector with isolation forest

As shown in Fig. 2B, this method employs a sliding time window to detect outliers in each sensor data segment within the time window. For each sensor data segment, we extracted features (e.g. statistical values such as the mean, variance, crest, and root-mean-square) and then concatenated the feature values to form a multivariate feature vector. See the SI (Features Extracted from Sensor Data) for the details of features extracted from sensor data. We extract a feature vector from each segment within the unlabeled sensor data using a sliding window and then train an isolation forest (22), which is an outlier detector that works well with high-volume data on the feature vectors. The isolation forest algorithm detects outliers by fusing binary decision trees, called isolation trees, which perform outlier detection independently. The isolation tree detects outliers based on the idea that, when a feature space is recursively split into small areas at random, an outlying feature vector is isolated in fewer splits because it is located far from the inliers in the feature space. As shown in Fig. 2C, we recursively split the feature space by randomly selecting a feature (dimension) and threshold value for each split. Information regarding each split is represented as a tree node in the isolation tree. In this example, the feature space was first split into two areas by the first feature *F1* using a threshold of 50. Therefore, as shown in the right portion of Fig. 2C, the root node of the isolation tree contains information about this split (i.e. the first feature *F1* and threshold 50). The left area was then split by the second feature *F2* using a threshold of five. In this case, the feature vector *O2* in the upper-left area was isolated by the second split. Therefore, as shown in the tree example, an isolated feature vector is assigned to the corresponding leaf node. When the number of unique feature vectors in an area becomes one, the random splitting of the area stops. In the isolation forest algorithm, multiple isolation trees are generated to perform robust outlier detection.

When we detect an outlier feature vector using the set of isolation trees, we first obtain the depth of a leaf node into which a feature vector of interest is classified for each isolation tree, and then compute the averaged depth over all isolation trees as an anomaly score of the feature vector. When the vector is isolated in fewer splits, the average depth (i.e. the anomaly score) decreases. A feature vector with an anomaly score lower than the threshold was detected as an outlier. Otherwise, it was regarded as an inlier.

### Constructing lightweight rare behavior detector with knowledge distillation

An isolation forest is generally composed of multiple trees; therefore, it is unfeasible to implement it on the MCU of a bio-logger with limited program memory as a rare-behavior detector. To address this issue, we employed knowledge distillation to train a lightweight rare-behavior detector using an outlier detector based on an isolation forest as a teacher model. We trained the lightweight rare-behavior detector to output the same results as the isolation forest, with a set of features used in the lightweight model identical to the teacher model. Specifically, we employed the outputs of the isolation forest (i.e. the outlier and inlier labels) for the unlabeled feature vectors as ground-truth labels to train the lightweight detector. Because the output of the isolation forest is a binary label (i.e. an outlier or inlier), a lightweight detector is implemented as a binary classifier. This study employs a decision tree as a lightweight rare-behavior detector because it has the following advantages when running on devices with limited program memory: (i) memory cost is low because it is implemented as a hierarchy of simple “if-then” rules, enabling the use of the remaining memory space for feature extraction algorithms; (ii) we can reduce computational costs by extracting only the necessary features from a sensor data segment within the hierarchy of the “if-then” rules (15). Each data segment is classified by following a single path through the tree from the root to leaf nodes, which represents the estimated class of the data segment (i.e. an outlier or inlier); thus, the MCU only needs to extract features as they are encountered in the path taken through the tree. This allows the MCU to run only a subset of the feature extraction processes for each data segment.

When constructing a decision tree using a standard decision-tree algorithm (23) that does not consider the memory costs of features, the trained tree can be memory inefficient. For example, when costly features are effective for a target task, many of the features are used in the tree. This results in an inefficient memory tree. Therefore, after randomly generating many decision trees while considering the memory costs of the features, we select a memory-efficient and high-accuracy decision tree and implement it in the MCU based on the idea of our previous study (15). To achieve this, we obtained the memory cost for calculating each feature in advance, which corresponded to the size of a compiled binary program from computer code that calculated the feature of interest from a sensor data segment. We then split the labeled data obtained from the teacher model (i.e. feature vectors with binary labels) into training and validation sets. Using the training set, multiple decision trees were randomly generated (Fig. 2D). In the decision-tree generation algorithm, the tree is built starting from the root node, with each node in the tree choosing (from among a set of features) one feature that can best split the training data passed to it into subsets that allow it to differentiate well between different target classes. A new child node is then created for each of the subsets of the training data output from that node, and this process is repeated recursively until certain stopping conditions are met (e.g. the subsets generated by a node reach a minimum size). Note that when we select a feature used in a node, the feature is selected from a set of randomly selected features, with the selection probability proportional to the inverse of the memory size of each feature. In other words, features with low memory costs tend to be used as node features in a tree, resulting in the generation of a memory-efficient tree. From the decision trees generated in this procedure, we selected a decision tree that fit the available program memory and yielded the best recognition performance for the validation set (Fig. 2D).

The selected decision tree is implemented on the MCU as a lightweight rare-behavior detector, and it detects outliers within a sensor data segment from low-cost sensors in real-time. Once an outlier is detected, the MCU activates its video camera for 5 min. See the SI (Tree Generation Algorithm) for details on tree generation. Our bio-loggers have a delay in video recording from sleep mode, making it difficult to capture outlier moments. As animals are considered to continue a specific behavioral mode, we used a long recording time, i.e. 5 min, to capture scenes related to the detected outliers.

### Procedure of performance evaluation of lightweight rare behavior detector

Our approach constructs a lightweight rare-behavior detector implemented on a bio-logger as a student model via knowledge distillation. Here, we explain the method for investigating the performance degradation of the student model in terms of outlier detection compared to the teacher model. Recall that the student model was trained on the labeled data (inlier and outlier labels) generated by the teacher model. Therefore, we split the labeled data generated by the teacher model into training and test sets. We constructed a student model using the training set and evaluated the student model using the test set.

### Field experiment of streaked shearwaters

The effectiveness of the proposed approach was evaluated using streaked shearwaters (*Calonectris leucomelas*) from a colony located on Awashima Island, Japan. We used unlabeled sensor data collected between 2017 August 4 and 28, 2018 August 20 and September 11, 2019 August 21 and 22, and 2020 August 21 and September 13 by using our bio-loggers on the same colony to train lightweight rare-behavior detectors. To capture a variety of behaviors, we constructed two different lightweight rare-behavior detectors: the first detected outliers in the three-axis acceleration data, and the second detected outliers in the water-depth sensor data. Acceleration-based detectors are expected to capture body movements caused by rare behaviors. In contrast, a water-depth-based detector is expected to capture rare behaviors occurring at the air–water interface. A simple approach relying on manually designed rules regarding water depth readings is unable to target rare behaviors because of (i) difficulties in selecting appropriate sensor data features and thresholds; and (ii) the difference in sensitivity of individual sensors, resulting in frequent or no activation of video recording. This is detailed below.

The input of the acceleration-based detector was a segment of three-axis acceleration data extracted from a sliding time window with a length of 1 s and no overlap. The input of the water-depth-based detector was a segment of water-depth data extracted from a sliding time window with a length of 3 s and no overlap. When an outlier was detected in a segment, the MCU activated the camera for five minutes. Table S2 shows all features extracted from the acceleration and water depth data, along with their memory costs.

The field experiment was conducted between 2022 August 26 and September 25. Our bio-loggers, equipped with lightweight rare-behavior detectors, were attached to 18 individuals (Fig. 1B and Table S3). The birds were captured in their nests by hand during the night, and the bio-loggers were mounted on the backs of the birds. Because the bio-loggers ran out of their batteries typically within 1- or 2 days due to video recording, we recaptured the birds whenever they returned to their nests. The bio-loggers were set to turn on at 5 AM and enter sleep mode at 6 PM because the camera is not capable of night photography. See the SI (Experimental Procedure of Streaked Shearwaters) for details on the field experiments.

During the field experiment, the bio-loggers collected ∼205 h of low-cost sensor data with an average of 14.6 h per individual. The number of 5-min videos captured by the acceleration-based and depth-based detectors was 54 and 22, respectively. Then, we manually analyzed the recorded videos using each type of detector and categorized the videos based on their captured behaviors. Consequently, videos were classified into three categories for each detector type. We discovered a novel behavior related to flying and head-shaking captured by the acceleration-based detector. Thus, we analyzed these events by processing the acceleration data collected in 2022 for deeper analysis. The details of the procedure are described in the SI (Analysis of Head-shaking during Flying).

## Results and discussion

We evaluated the proposed approach by running lightweight rare-behavior detectors on the bio-loggers attached to seabirds. In addition, we quantitatively evaluated the performance of the lightweight rare-behavior detector using the outputs of the teacher model as the ground truth.

### Analysis of videos captured by acceleration-based rare-behavior detector

The scenes in the videos collected by the acceleration-based detector were classified into three categories: (i) 35 were triggered by the action of the birds shaking their heads immediately after they took off from the sea surface, (ii) five were triggered by the birds shaking their heads while floating on the sea surface, and (iii) seven were triggered by sudden intense body movements during preening. The remaining videos did not fall under any category.

**Fig. 3.**
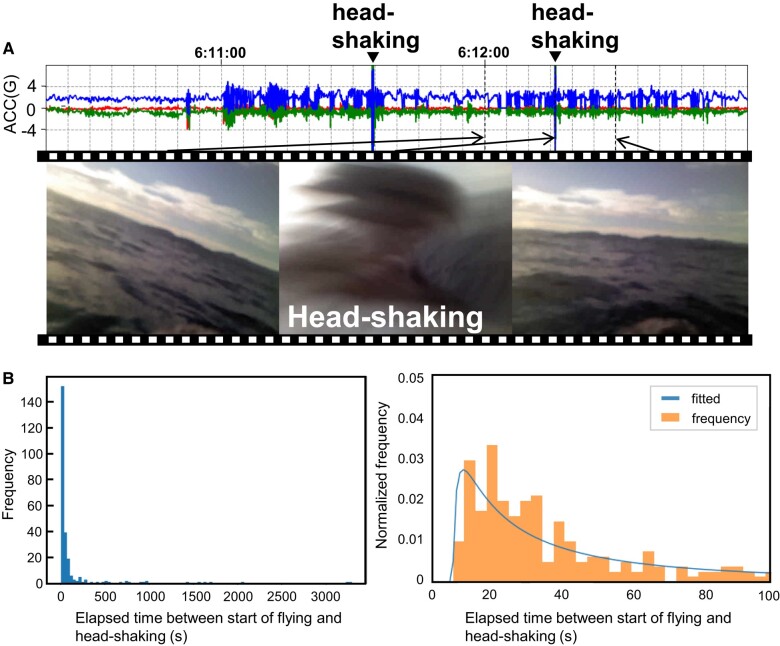
Results of experiment using acceleration-based rare-behavior detector. A) Examples of sensor data and video frames taken when the seabird shakes its head during flight. B) Histogram of elapsed time between the start of flight and head-shaking. The frequency of head-shaking has been fitted with the log-normal distribution (mean: 101.2, standard deviation: 282.3).

In the first category, the head-shaking behavior during flight was detected as outliers (Fig. 3A and Movie S2). Specifically, because the birds seemed to shake their heads just after they took off from the sea surface, we measured the elapsed time between the start time of the flying behavior and the head-shaking action. Figure 3B shows a histogram of the measured elapsed times, suggesting that head-shaking actions were performed intensively at the beginning of flying behavior. Note that, as mentioned above, although the bio-loggers also collected videos capturing head-shaking while floating on the sea surface, the number of videos was much smaller than those capturing head-shaking during flight.

Seabirds are typically exposed to high-salinity seawater. Their nasal salt glands filter excess salt from the bloodstream to maintain a constant salt concentration (i.e. osmoregulation) (24). In some seabirds (such as penguins), it is well-known that they remove nasal salt gland fluids, as well as water, food, dirt, and snow from their heads and bills by head-shaking (25). They also sneeze to remove nasal salt gland fluid (25). Our results showed that streaked shearwaters often engaged in head-shaking or sneezing soon after take-off. Streaked shearwaters may have performed these behaviors to remove their nasal salt gland fluids during flight, after floating, after bathing, or after foraging at sea for some time. Head-shaking in streaked shearwaters may also remove external materials, including water. Moreover, the removal of nasal salt gland fluids and other external materials (including water) decreases total body mass, which could then increase flight efficiency (26). While head-shaking in seabirds consumes energy to a certain degree (27), frequent head-shaking on leaving the water surface might impact the behavioral time budget during their foraging trips.

Refer to the SI (Analysis of Preening Behavior and Head-shaking while Floating) for an analysis of the second and third video categories.

### Analysis of videos captured by water-depth-based rare-behavior detector

**Fig. 4.**
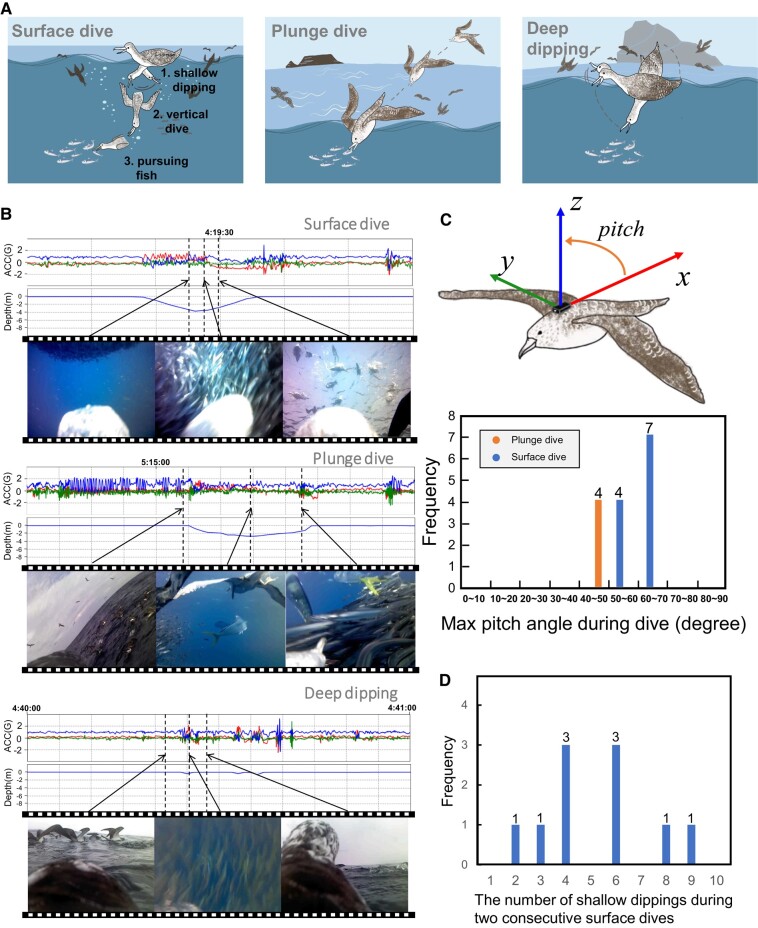
Results of experiment using water-depth-based rare-behavior detector. A) Observed three categories of foraging behaviors. B) Examples of sensor data and frames taken during surface dive, plunge dive, and deep dipping. C) Acceleration sensor placement, pitch angle, and histogram of pitch angles in surface and plunge dives. See the SI (Analysis of Diving Behavior) for the manner of pitch angle calculation. D) Histogram of the number of shallow dips during two consecutive surface dives.

The scenes of the videos collected by the water-depth-based detector were roughly classified into three categories (Fig. 4A): (i) six videos were triggered by the birds deep diving into the sea after floating on the sea surface (top panel of Fig. 4B and Movie S1), (ii) two videos were triggered by the birds diving into the sea during a low-altitude flight (middle panel of Fig. 4B and Movie S1), and (iii) two videos were triggered by the birds deeply dipping their heads underwater while floating on the sea surface (bottom panel of Fig. 4B and Movie S1). Because the frames of these videos contain fish, they were considered to be recorded during foraging behaviors. Here, we define these behaviors as surface dive, plunge dive, and deep dipping for the first, second, and third categories, respectively. The remaining videos did not fall under any category.

Recording videos of foraging is generally difficult because the frequency of foraging activities is quite low (28). Thus, the videos of foraging collected by our bio-loggers may contain novel behaviors related to foraging that we have not yet identified. Our bio-loggers recorded 50 min of foraging videos, enabling us to deeply analyze foraging scenes in conjunction with their associated sensor data.

As shown in the top panel of Fig. 4B, during the behavior of the first video category (i.e. the surface dive), the water depth sensor data significantly changed and were identified as outliers. During the surface dive, the seabirds appeared to dive into the sea vertically in many of the videos (Fig. 4C), moved horizontally while pursuing the fish school, and finally rose vertically to the surface. Importantly, our observations of surface dive behaviors suggested that the seabirds dipped their heads underwater several times before the surface dive, which is defined as shallow dipping behavior in this study (Movie S1). Figure 4D shows a histogram of the number of shallow dips performed during two consecutive surface dives (i.e. between the end of the former dive and the start of the latter dive) counted by watching the videos, suggesting that the seabirds performed shallow dips ∼5 times before the surface dive. Therefore, seabirds are considered to apply a foraging strategy in which they first confirm the location of a fish school by consecutive shallow dips and then dive vertically when fish schools are within the range of their dives or are anticipated to enter the range (left panel of Fig. 4A). In general, shearwater species are well-known for floating on the water surface and dipping their head into the water before diving (28–31), which is often interpreted as a means of locating prey before submersion (29, 30). Performing shallow dipping to confirm the location of fish schools before diving can reduce energy consumption and increase the probability of successful foraging. Given that underwater visibility influences the foraging behavior and success of seabirds (32), the number of dips might vary based on weather conditions and water turbidity. Streaked shearwaters might employ surface dives, plunge dives, and deep dipping in relation to the accessibility of prey.

Streaked shearwaters are known to perform shallow dives. For example, Oka (33) reported that the birds dived at least 5 min, and Matsumoto et al. (34) reported that the birds dived 6 min and 18 s at maximum. Our data showed that the birds dived 6.1 min and 14.7 s at maximum, similar to the previous reports. The videos recorded by our bio-loggers enable us to deeply analyze the foraging behavior of streaked shearwaters. As Fig. S5 suggests, the maximum depth can depend on the category of foraging behaviors, i.e. surface dive vs. plunge dive. The maximum depth of the plunge dive seems shallower than that of the surface dive. This may be because the seabirds attempted to capture fish close to the sea surface by the plunge dive due to the limited visibility of fish schools during flying.

For the analysis of the second and third video categories (i.e. plunge dive and deep dipping), refer to the SI (Analysis of Diving Behavior).

### Outlier detection performance of lightweight rare behavior detector

**Fig. 5.**
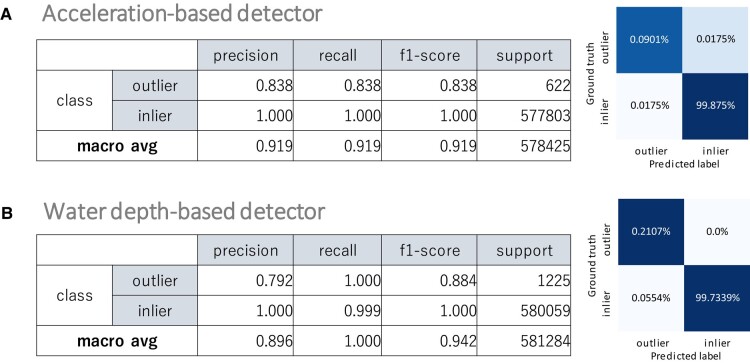
Experimental results of outlier detection performance. A) Outlier detection performance and confusion matrix of the acceleration-based lightweight rare-behavior detector. B) Outlier detection performance and confusion matrix of the water-depth-based lightweight rare-behavior detector.

We evaluated the performance of the lightweight detectors according to the procedure described in the *Procedure of Performance Evaluation of Lightweight Rare-Behavior Detector* section. Figure 5A and B show the outlier detection performance of the lightweight detectors calculated based on the feature vectors in the test set. Although the dataset was imbalanced, the average F1-score was higher than 90%, and the F1-score for the outlier class was maintained at 80%. The water-depth-based detector achieved 100% recall for the outlier class, meaning that the detector could record outlying events without missing any events.

### Ability of finding unknown behavior pattern

We evaluated the ability of our bio-loggers in terms of finding unknown behavior patterns. As mentioned above, the acceleration-based detector captured 54 videos. Thirty-five of them captured scenes related to the head-shaking behavior after take-off, relevant to the novel findings of this study. Five videos captured the head-shaking behavior while floating on the sea surface. Interestingly, the interval of the head-shaking actions gradually shortened, as mentioned in the SI. To the best of our knowledge, no study focuses on the interval of the head-shaking actions by seabirds. Seven videos captured scenes regarding intense body movements during preening, and the scenes did not contain novel behaviors. Although the remaining seven videos seem to capture head-shaking behaviors because the sensor data that triggered video-recording are similar to those of the head-shaking actions, they were hard to analyze due to occlusions or delays in video-recording. As a result, out of the 54 recorded videos, 35 videos (64.8%) captured scenes regarding unknown behavior patterns. Twelve videos (5 + 7 = 12; 22.2%) possibly captured scenes related to unknown behavior patterns. The remaining seven videos (13.0%) seem to capture only trivial behaviors.

The depth-based detector recorded 22 videos. Out of the 22 videos, 10 videos (45.5%) captured scenes regarding foraging behaviors. Although the foraging behaviors are known, the comprehensive analysis based on the 10 videos revealed the strategy of foraging, also a finding of this study. The remaining 12 videos (54.5%) were hard to analyze due to occlusions. Because many of these videos were recorded when the seabirds were on the sea surface, the scenes were usually occluded by the heads and necks of the seabirds.

### Setting of experimental parameters and sensor data features

Our method relies on several manually selected parameters/features. For example, the size of a sliding window is manually selected as one second in the acceleration-based detectors. However, the optimal window size can depend on the movement characteristics of animals, necessitating a longer time window for slow-moving species. In addition, selection of sensor data features greatly affects rare behaviors to be detected. However, selecting optimal parameters/features to use before experiments is difficult because we have no prior knowledge about rare behaviors.

The parameters/features should also be determined according to memory/computation costs. Our experiment employed almost the same setting as that of our prior study (15) because we used the same hardware. Note that we used a 3 s time window for water depth data collected at 1 Hz in order to compute meaningful features. When applying our method to other species, we should select parameters/features to ensure real-time functionality of the devices. In addition, because we have no prior knowledge about rare behaviors, it is recommended to refer to parameters/features used in prior behavior recognition studies for the target species, enabling us to adapt to the general behavioral pattern/speed of the species.

### Difference in sample size among individuals in rare behaviors

When the number of videos capturing a specific behavior is few or zero in some individuals, we should investigate the reasons for it. It is considered that sensor signals for the same behavior can be different in different individual animals when the behavior is considered to be diverse among individuals (35). In addition, it is possible that the behavior did not occur during the experiment period due to its rarity. Furthermore, the behavior is considered to occur in individuals with specific properties, e.g. male-specific behaviors. In our study using the acceleration-based detectors, the head-shaking behavior was observed in all the individuals, indicating its limited diversity among individuals (average: 1.12 and standard deviation: 0.44, the number of observed head-shaking actions per hour except individuals with corrupted bio-loggers). In our study based on the depth-based detectors, although the identified foraging strategies such as that found in the surface dive were not observed in all the individuals, it is due to the rarity of the event because the recorded videos captured the behavior conducted by many other surrounding individuals (Movie S1).

### Significance and implications

While innovations in sensing technologies have enabled the collection of big behavioral data from low-cost sensors, such as accelerometers, rare behaviors are buried in big data (36). Although unsupervised learning techniques have been adopted for animal behavior data (16, 17), these studies mainly focus on clustering techniques in an offline manner to identify frequent behavior patterns in a given data, making it difficult to find rare behaviors. Besides, innovations in microcomputers enable on-board processing of sensor data, e.g. on-board behavior classification for video-recording a target behavior (15) and for precise travel distance prediction (37).

Based on on-device outlier detection techniques, our bio-logger automatically detects rare events and records videos that facilitate the understanding of rare events in conjunction with low-cost sensor data. Although methods for on-device outlier detection have been studied in the field of information science, many recent approaches are based on deep learning models (e.g. autoencoders) (20) and are not assumed to run on mobile devices with limited batteries (e.g. animal-borne devices). Many classic outlier detection methods, such as those based on the local outlier factor (LoF) (18), are also inefficient because they require a distance calculation between an incoming feature vector and each unlabeled feature vector. Although a one-class support vector machine (SVM) (19) is also typically used in outlier detection tasks, it is computationally inefficient because all the features used should be computed in advance.

Various sampling strategies for bio-logging devices have been used, such as acceleration-, water depth-, and illumination level-based triggering mechanisms (9–14) to target specific animal behaviors based on the prior domain knowledge of researchers (i.e. handcrafted rules of sensor data features). In contrast, our approach automatically captures unusual events; thus, scenes of behaviors that researchers have not anticipated in advance are possibly recorded. A case in point is the head-shaking behavior recorded by an acceleration-based detector during flight. Our analysis revealed that the streaked shearwaters performed head-shaking behaviors at the beginning of flight. This suggests that they attempted to remove external materials to increase the efficiency of subsequent flights, as a component of the fit-for-flight hypothesis that reduces body weight at the start of flight (38).

The water-depth-based detector can capture videos of rare behaviors at the air–water interface, which is pivotal for understanding foraging tactics and interactions between seabirds and fish. Our video analysis revealed an efficient foraging strategy for streaked shearwaters by confirming the location of a fish school via consecutive shallow dips before diving. As mentioned in the *Field Experiment of Streaked Shearwaters* section, it is difficult for the handcrafted rule-based approach to target infrequent behaviors at the air–water interface because of difficulties in selecting appropriate features and thresholds and the difference in sensitivity of individual sensors. Readings of water depth sensors can capture a variety of trivial behaviors at the air–water interface (e.g. preening), as well as contain noise associated with wave actions. Therefore, the sensor data difference between the infrequent and frequent behaviors at the air–water interface is not apparent (Fig. S3). Designing rules to capture only infrequent behaviors is difficult for researchers without a great deal of experience in machine learning, even when prior knowledge of researchers is available. In contrast, our approach enables us to automatically capture rare behaviors without prior knowledge from the researchers. In addition, the difference in sensitivity and sensor data drift of individual depth sensors makes it difficult to apply a rule-based approach that relies on manual thresholds. Because of the above issues, different thresholds should be carefully set for different bio-loggers to detect infrequent behaviors at the air–water interface. At this interface, the magnitude of the depth data is inherently small, which results in huge set-up/calibration costs when many bio-loggers are deployed. In contrast, the lightweight detector seems to be automatically trained to be robust against differences because it is mainly composed of water depth features related to data fluctuations (i.e. the variance and mean cross), which may not be significantly affected by differences in sensitivity and drift. It is important to note that the water-depth-based detector can capture rare behaviors at the air–water interface in an unsupervised manner (i.e. without any configurations or setups based on the prior knowledge of researchers).

To the best of our knowledge, this is the first study to employ unsupervised learning for efficient bio-logging. We believe that our approach can also be used to acquire labeled data for supervised learning. For example, when a researcher requires additional data on a novel behavior discovered by our method, we can employ a supervised method (15, 39) trained on the data collected by our method as the labeled data. The main limitation of the proposed approach is that the novelty of the rare behaviors captured by our approach is not assured because the approach relies completely on unsupervised learning. A hybrid supervised/unsupervised approach is a possible future research direction for smarter bio-logging, e.g. introducing a blacklist of specific rare behaviors to capture via supervised learning.

Our method overcomes the primary limitation of animal-borne cameras: their short battery life (8, 40) and can be applied across a wide range of species in three primary applications. First, our approach facilitates the documentation of novel behaviors in animal populations, either at their emergence or immediately thereafter, propagating through social learning, such as the sweet-potato washing observed in Japanese monkeys (41) and the innovation of waste bin opening by wild birds (42). Second, our method captures instantaneous behaviors that are often overlooked. Examples include how wild birds react to sudden environmental changes such as aerial turbulence (43), how omnivorous animals consume rare prey items (e.g. fish- and krill-eating penguins occasionally feed on jellyfish (13)), and brief social interactions such as kleptoparasitism by gulls (15). Finally, our technique aims to record behaviors that are challenging to observe but hold significant importance for understanding an animal's life-history, like underwater mating of marine animals, or the causes of deaths in the wild (44).

As part of our future study, we plan to apply our approach to a variety of species and investigate how our approach is effective in the above three applications. In addition to the acceleration and depth sensors we used as behavioral triggers, a variety of other sensors can be incorporated in the future. For example, it would be feasible to record movies of behavior and the surrounding environment when abnormalities (i.e. outliers) in internal conditions are detected, such as through electrocardiograms (45) or electroencephalograms (46).

Many animals spend most of their time on trivial behaviors. Our approach enabled us to record nontrivial behaviors without the supervision of researchers. It is impractical for researchers to supervise the behavior of wild animals where direct fine-grained observation is difficult. The proposed approach is also useful for researchers with limited knowledge of machine learning because a rare-behavior detector can be automatically trained on unlabeled data. Subsequently, the recorded videos associated with the sensor data were used to propose a hypothesis and investigate the rare behaviors identified from the recorded videos and sensor data. We believe that this study paves the way for the creation of a small naturalist on a bio-logger using data-driven AI.

## Supplementary Material

pgad447_Supplementary_Data

## Data Availability

The sensor data collected in our field experiment and the unlabeled feature vectors used to construct the teacher and student detectors are included in Ref. (48). Python (v.3.8.5) and scikit-learn (v.0.23.2) (47) were used to implement teacher and student detectors. The codes run on the bio-logger were written in C++ (Arduino v.1.8.19). The codes for our bio-loggers are included in Ref. (48).

## Notes

a The main difference between supervised and unsupervised learning is the use of labeled datasets. In supervised learning, the algorithm learns to map input data to output data, i.e. labels, based on a set of labeled examples. In contrast, in unsupervised learning, the algorithm learns to identify patterns in the input data without any labeled examples.

b Knowledge distillation is a machine learning process that transfers knowledge from a large teacher model to a smaller student model. The teacher model is assumed to be trained in advance, and then the student model is trained so that it emulates the teacher model.
