# Supplementary material for: Automatic recording of rare behaviors of wild animals using video bio-loggers with on-board light-weight outlier detector
Source: PNAS Nexus. 2024 Jan 16;3(1):pgad447. doi: 10.1093/pnasnexus/pgad447 (PMC10791039; doi:10.1093/pnasnexus/pgad447)
Supplement: pgad447_Supplementary_Data [file pgad447_supplementary_data.zip › PNASNEXUS-PNASNEXUS-2023-00540RR-s01.pdf]

Supporting Information for

Automatic recording of rare behaviors of wild animals using video bio-loggers with on-board light-weight outlier detector

Kei Tanigaki, Ryoma Otsuka, Aiyi Li, Yota Hatano, Yuanzhou Wei, Shiho Koyama, Ken Yoda, Takuya Maekawa

maekawa@ist.osaka-u.ac.jp

This PDF file includes:

Supporting text
Figs. S1 to S6
Tables S1 to S3
Legends for Movies S1 to S2
Legends for Dataset S1 to S2
SI References

Other supporting materials for this manuscript include the following:

Movies S1 to S2
Datasets S1 to S2

Supporting Information Text

Table S1. Specifications of bio-loggers used in this study

Sensor type	Model	Sampling rate (Hz)
Accelerometer	BMX-055	31
Water depth sensor	MS583730BA01-50	1
GPS	CAM-M8Q	1/60
Magnetometer	BMX-055	1
Temperature sensor	MS583730BA01-50	1
Illumination sensor	BH1721FVC	1

Features Extracted from Sensor Data

Table S2 shows acceleration and water depth features used in this study and their memory costs. The features are calculated from a time window of time-series data. The mean cross is the count of how many times two adjacent values cross over the mean value within a time window. For example, time-series data $\mathbf{x} = [0, 2, 2, 1]$ with length 5 is given and the mean is calculated as 1.25. Between time 1 and time 2, the values, i.e., 0 and 2, cross over the mean. In addition, between time 3 and 4, the values, i.e., 2 and 1, cross over the mean again. Therefore, the mean cross is calculated as 2 in the time-series data. The one cross is the count of how many times two adjacent values cross over 1. The RMS shows the root mean square within a window. The mean difference is the sum of the difference between the mean and each data point.

Table S2. Features extracted from acceleration and water depth data and their memory costs (bytes)

ACC feature	Cost	Water depth feature	Cost
mean	114	mean	164
mean cross	222	mean difference	212
one cross	204	mean cross	286
variance	256	variance	386
energy	134	energy	200
RMS	138	RMS	204
kurtosis	888	kurtosis	922
crest	390	crest	418

Tree Generation Algorithm

We generated decision trees (lightweight rare behavior detectors) with low memory costs by using feature vectors with outlier/inlier labels created by the original rare behavior detector (isolation forest) as training data. Here we explain the procedure in detail. We first selected a validation set that is used to assess the performance of decision trees to generate. We conducted leave-one-bird-out cross validation to generate a standard decision tree in each validation by using the scikit-learn library (1) and then tested the decision tree on feature vectors from a test bird to calculate the F1-score for the test bird. We selected top-two test birds closest to the median in their F1-scores as representative birds. The feature vectors from the two selected birds were used as the validation set. The feature vectors from the remaining birds were used as the training set. Then we generated decision trees by using the training set based on the procedures described in the main text. When we choose a feature used in a node, the feature was chosen from a set of randomly selected features from all features. The selection probability for a feature is inversely proportional to its memory cost.

Experimental Procedure of Streaked Shearwaters

The bio-loggers were attached to the bird's back by taping them to the bird's feathers using waterproof tape. Sample size of the birds was determined by the availability of bio-loggers. See (2) for general information about the field experiments. Table S3 shows information on the streaked shearwaters. The table also shows the number of videos captured by the acceleration-based and water-depth-based detectors.

To record acceleration- and depth-based videos evenly, each bio-logger was configured so that it records up to six acceleration-based videos. This is because our preliminary experiment revealed that outliers in water depth data sometimes occur within short intervals, resulting in fewer video recordings than acceleration-based videos. In the water-depth-based detector, a threshold used to detect outliers based on anomaly scores was determined so that 0.02% of all the feature vectors extracted from the unlabeled data become outliers, and the number of isolation trees was 100, which are the default parameters of the scikit-learn library (1). In the acceleration-based detector, we used 0.01% for the threshold of anomaly scores because of the same reason for introducing the configuration of the maximum number of acceleration-based videos.

Table S3. Information about individual seabirds used in the experiment and the numbers of videos recorded by the acceleration and water-depth-based detectors. The sexes of the birds were determined based on their vocalizations (3).

Identifier	Sex	Body mass (g)	# Acc videos	# Water depth videos	Note
1	Female	535	5	0	
2	Male	600	5	1	
3	Female	520	1	0	
4	Male	565	5	4	
5	Female	505	5	1	
6	Female	530	0	0	Bio-logger lost
7	Female	475	0	0	Video storage broken
8	Male	620	5	1	
9	Female	455	5	1	
10	Male	590	0	0	Camera broken
11	Male	580	5	7	
12	Male	600	0	0	Bio-logger lost
13	Female	495	5	0	
14	Female	515	2	1	
15	Female	500	5	3	
16	Female	580	0	0	Sensor data storage broken
17	Male	590	6	3	
18	Male	605	0	0	Bio-logger lost

Analysis of Head-shaking during Flying

Here we explain the way of measuring the elapsed time between the start of flying and head-shaking actions detected by the detector. We employed a sliding time window and a simple thresholding method to determine the start time of the flying corresponding to a head-shaking action of interest. The time window was slid backward from the occurrence time of the head-shaking action, and the magnitude within the window was calculated. We used a time window with the length of 1 s and the stride length of $\frac{1}{3}$ s. Once the calculated magnitude became smaller than a threshold, we decided that the time corresponds to the start time of the flying. Note that we manually double-checked the detected start times because waveforms corresponding to flying and head-shaking are apparent.

Analysis of Preening Behavior and Head-shaking while Floating

Our bio-loggers recorded intense body movements during floating on sea surface when the seabirds groomed their breast feathers. As shown in Figure S1, short segments with high amplitudes corresponding to preening actions are intermittently observed. We found two major occurrence patterns of the preening actions in terms of the occurrence intervals. The left part of Figure S1 shows the first occurrence pattern where the interval is uniform. In contrast, the right part of Figure S1 shows the second occurrence pattern where the interval gradually shortens, and then the birds stop the preening actions. Although we could not understand the reason behind the decrease in the interval, the frequency of the first and second occurrence patterns are almost identical.

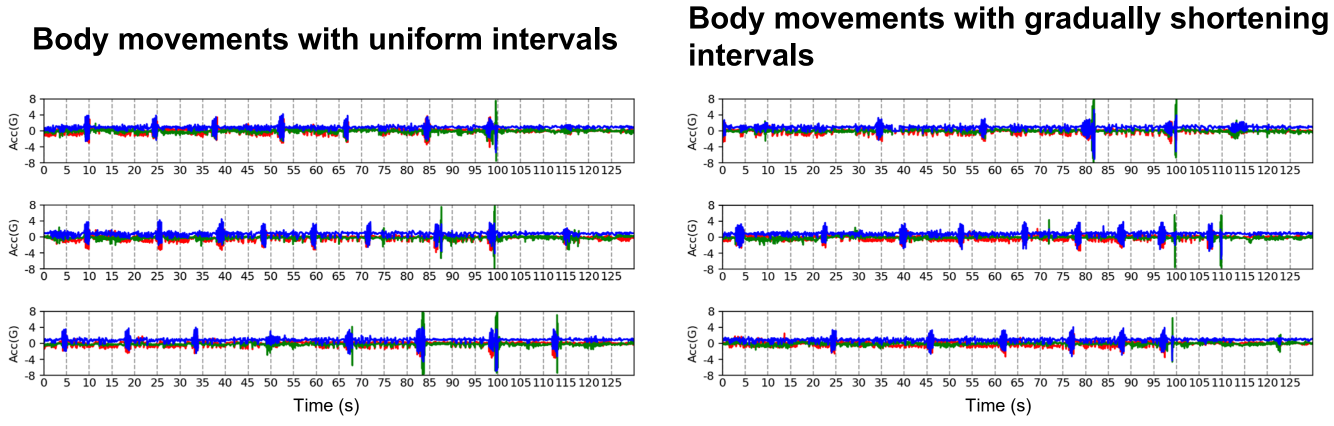

Fig. S1. Acceleration data when the seabirds performed intense body movements while preening

Our bio-loggers also recorded head-shaking actions while floating as shown in Figure S2. Although the purpose of the head-shaking is expected to be identical to that of the head-shaking while flying, the frequency of the head-shaking while

floating was much lower than that of the head-shaking while flying.

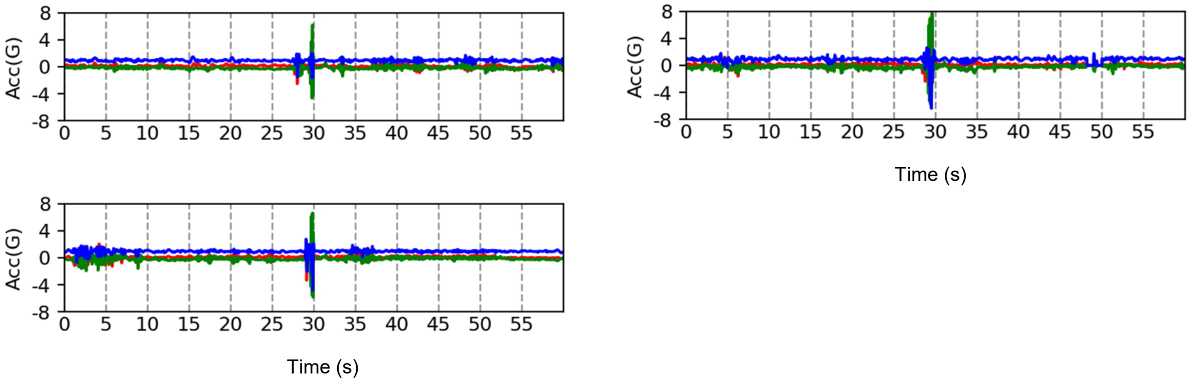

Fig. S2. Acceleration data when the seabirds performed head-shaking while floating

Analysis of Diving Behavior

Here we explain the way of calculating the pitch angle from accelerometer data. Because the accelerometer data contains effects of body actions by the birds, we removed sensor data points when their magnitude are larger than 1.1 G or smaller than 0.9 G and then interpolated the removed data points by the linear interpolation for each axis. After calculating rolling average of the interpolated data with the window length of 0.5 s for each axis according to (4), we calculated the pitch angle for each time step by $\arctan(a_x/a_z)$, where a_x and a_z are the x- and z-axis acceleration values, respectively. When we calculated the pitch angle, we assumed that the body is not inclined to lean to the left or right.

As shown in Figure 4c in the main text, the pitch angle for the surface dive seems to be larger than that for the plunge dive. We investigated the statistical difference in the pitch angle between the surface dive and plunge dive using a linear model and maximum likelihood estimation although we could not consider individual factors because plunge dives were observed only on a single individual. A test was two-tailed and the significance level was set at 0.05. The number of data points for the surface dive is 10 and the number of data points for the plunge dive is four. We used the lm function of R (v. 4.1.1) (5) for the analysis. The investigation revealed that the pitch angle significantly differs between the surface dive and plunge dive (coefficient=23.21, 95% confidence intervals=15.83 - 30.59, standard error=3.39, $p = 1.77 \times 10^{-5}$ (Wald test), degree of freedom=12).

The water-depth-based detector could record three types behaviors at the air-water interface, surface dive, plunge dive, and deep dipping, enabling to deeply analyze foraging strategy of the streaked shearwaters. As shown in the lower panel of Fig. 4B and Movie S1, the streaked shearwaters tried to capture fish in shallow water by deep dipping. In contrast, as shown in the middle panel of Fig. 4b, the streaked shearwaters captured fish at mid-depth by plunge dive. They indicate that the streaked shearwaters change the foraging strategy depending on the depth of a fish school.

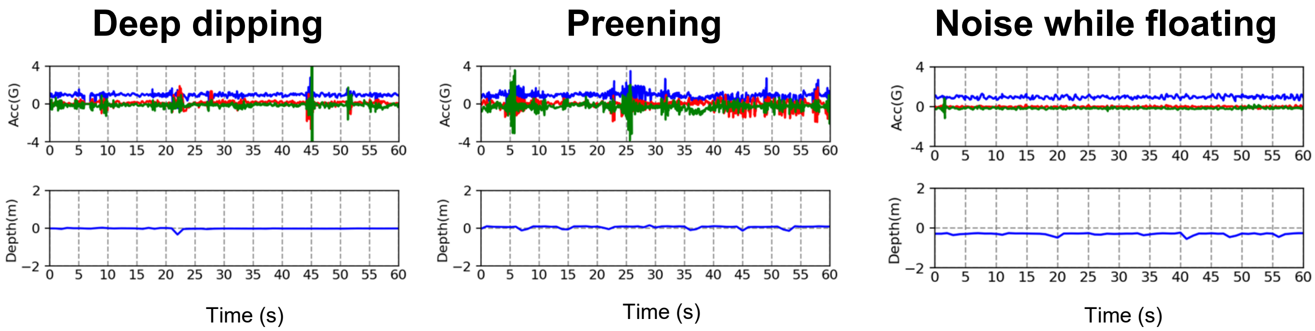

Fig. S3. Acceleration and water depth data observed at the air-water interface

Application to Black-tailed Gulls

To show the generalizability of our approach, we apply our method to acceleration data of black-tailed gulls (*Larus crassirostris*) from a colony located on Kabushima Island near Hachinohe City, Japan, collected in our prior study (6). The acceleration data were collected by our devices from 28 birds in May 2018 and 2019. The total duration of the collected data is 419 hours. The bio-loggers were configured to record five-minute videos randomly or when foraging behaviors were detected by supervised learning based on acceleration data (6). The number of recorded videos is 743.

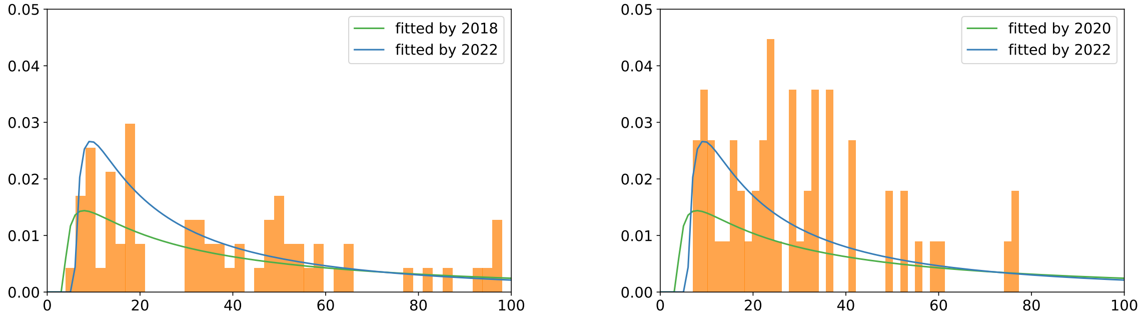

Fig. S4. Histograms of elapsed time between the start of flying and head-shaking in 2018 and 2020

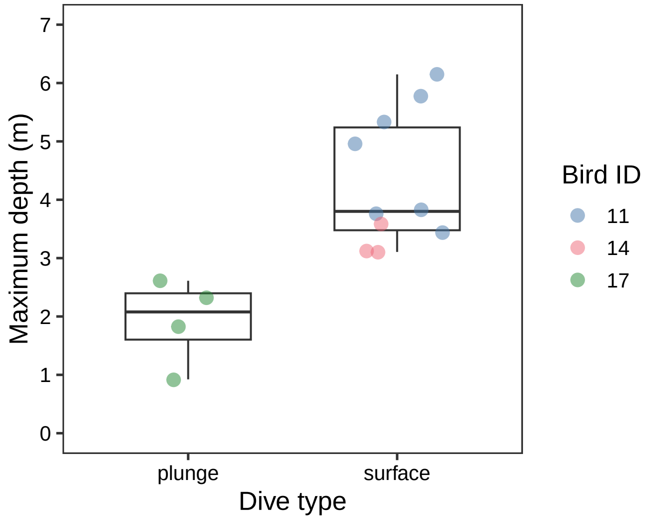

Fig. S5. Relationship between type of foraging dive behavior and maximum depth

We applied our rare behavior detector to the acceleration data and categorized the detected outliers captured by the videos. As a result, we could obtain various behavior categories detected by the rare behavior detector: 24 head-shaking events (acceleration data segments) during flying, 6 preening events, 4 foraging events (including 3 possible foraging events), 3 take-off events, 3 circling events, and 3 flapping on the ground. 5 events did not fall under the above categories. The remaining 34 events were hard to analyze due to occlusions.

The rare behavior detector detected 4 foraging events, and a video corresponding to one of the detected events contained an appearance of fish. The prior supervised method could capture only one foraging video containing the appearance of fish. Surprisingly, the rare behavior detector could detect the scene without using labeled training data.

Interestingly, the head-shaking behaviors during flying were also detected in the experiment of the black-tailed gulls. Similar to the analysis of the streaked shearwaters, we also calculated the elapsed time between the start time of the flying behavior and the shaking action. Fig. S6 shows that the black-tailed gulls also tend to perform the action just after the take-off. These results support our hypothesis that the streaked shearwaters perform this action to remove external materials in order to improve flight efficiency.

The remaining videos did not seem to contain new findings. As a result, 29.3% of videos (24/82) seem to capture scenes of unknown behavior patterns. As a part of our future work, we thoroughly analyze the remaining videos to see if the new findings are hidden.

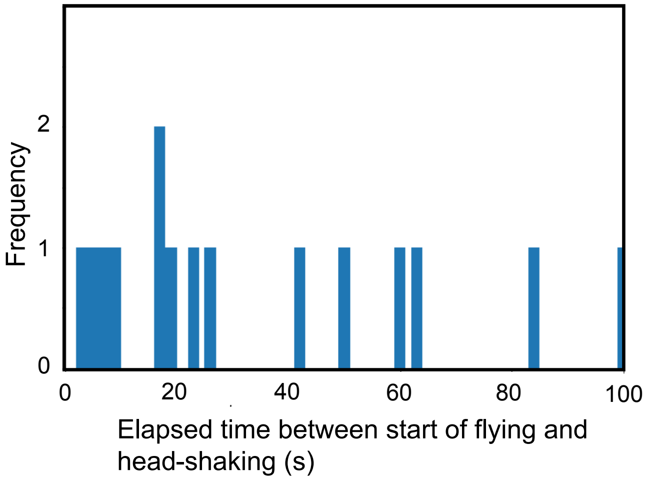

Fig. S6. Histogram of elapsed time between the start of flight and head-shaking (black-tailed gulls)

Movie S1. Surface dive, plunge dive, and deep dipping recorded by water-depth-based rare behavior detector

Movie S2. Head-shaking while flying, head-shaking while floating and, intense body movements while preening recorded by acceleration-based rare behavior detector

SI Dataset S1 (FigureData.xlsx)
Source data of Fig. 3 and Fig. 4

SI Dataset S2 (SupplementaryData.zip)
Sensor data collected by our field experiment and unlabeled feature vectors used to construct teacher and student detectors

References

1. F Pedregosa, et al., Scikit-learn: Machine learning in python. *J. machine Learn. research* **12**, 2825–2830 (2011).
2. S Matsumoto, T Yamamoto, M Yamamoto, CB Zavalaga, K Yoda, Sex-related differences in the foraging movement of streaked shearwaters *Calonectris leucomelas* breeding on awashima island in the sea of japan. *Ornithol. Sci.* **16**, 23–32 (2017).
3. H Arima, N Oka, Y Baba, H Sugawa, T Ota, Gender Identification by Calls and Body Size of the Streaked Shearwater Examined by CHD Genes. *Ornithol. Sci.* **13**, 9 – 17 (2014).
4. EL Shepard, et al., Identification of animal movement patterns using tri-axial accelerometry. *Endangered species research* **10**, 47–60 (2008).
5. R Core Team, R: A language and environment for statistical computing. (2021).
6. J Korpela, et al., Machine learning enables improved runtime and precision for bio-loggers on seabirds. *Commun. Biol.* **3** (2020).
