# Supplementary figures and images for: Automatic recording of rare behaviors of wild animals using video bio-loggers with on-board light-weight outlier detector
Source: PNAS Nexus. 2024 Jan 16;3(1):pgad447. doi: 10.1093/pnasnexus/pgad447 (PMC10791039; doi:10.1093/pnasnexus/pgad447)

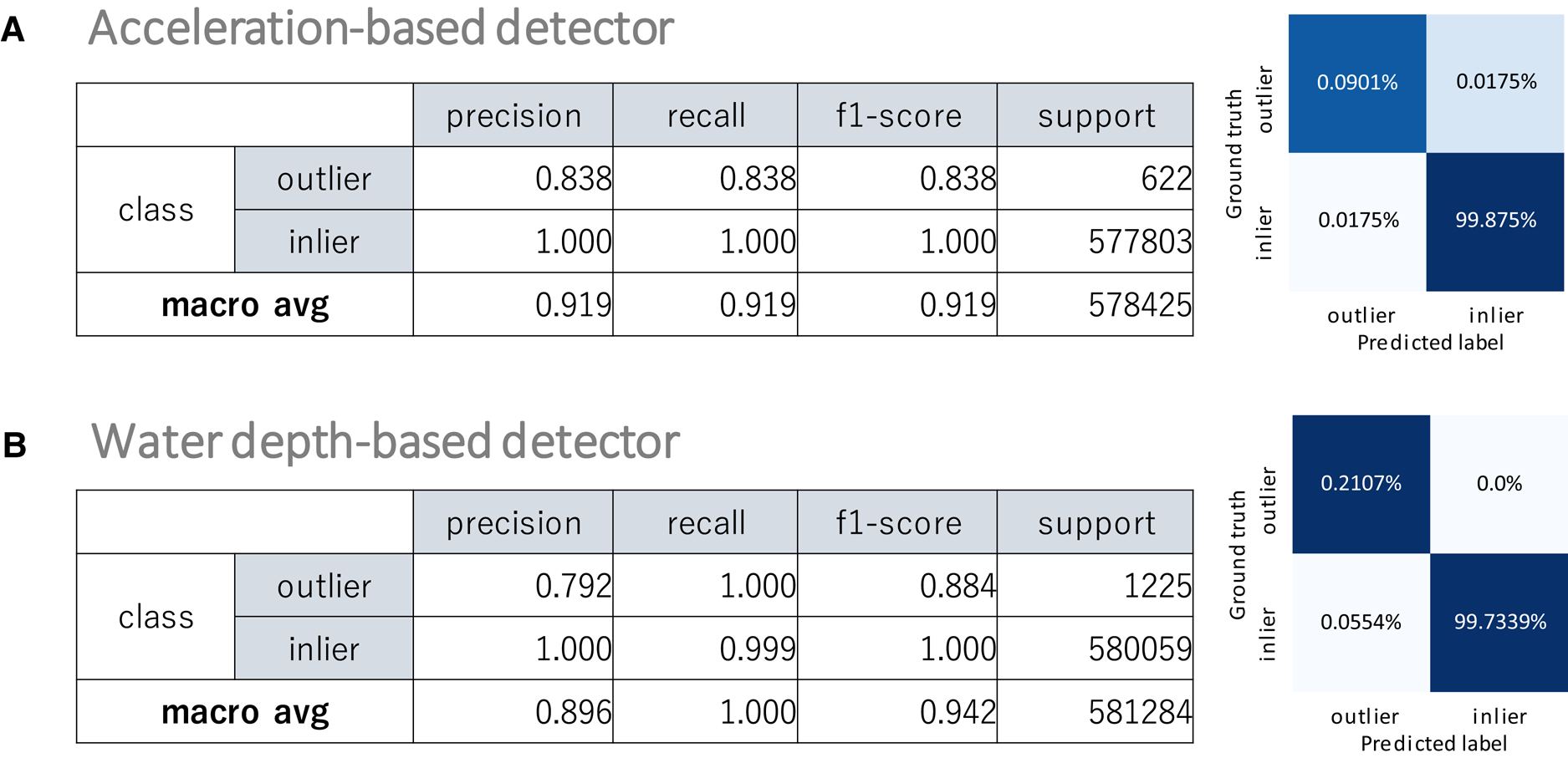

Supplement: pgad447_Supplementary_Data [file pgad447_supplementary_data.zip › pgad447f5.tif]
